# Strain-Induced Modifications of Thin Film Silicon Membranes Through Physical Bending

**DOI:** 10.3390/ma18102335

**Published:** 2025-05-17

**Authors:** Eleni Margariti, Jochen Bruckbauer, Aimo Winkelmann, Benoit Guilhabert, Naresh-Kumar Gunasekar, Carol Trager-Cowan, Robert Martin, Michael Strain

**Affiliations:** 1 Institute of Photonics, Department of Physics, Scottish Universities Physics Alliance (SUPA), University of Strathclyde, Glasgow G1 1RD, UK; benoit.guilhabert@strath.ac.uk (B.G.); michael.strain@strath.ac.uk (M.S.); 2 Department of Physics, Scottish Universities Physics Alliance (SUPA), University of Strathclyde, Glasgow G1 1XQ, UK; jochen.bruckbauer@strath.ac.uk (J.B.); aimo.winkelmann@strath.ac.uk (A.W.); gunasekarn@cardiff.ac.uk (N.-K.G.); c.trager-cowan@strath.ac.uk (C.T.-C.); r.w.martin@strath.ac.uk (R.M.); 3 Academic Centre for Materials and Nanotechnology, AGH University of Krakow, 30-059 Kraków, Poland; 4 Institute for Compound Semiconductors, School of Physics and Astronomy, Cardiff University, Cardiff CF10 3AX, UK

**Keywords:** silicon photonics, EBSD, micro-transfer printing, strain engineering, FEA model, mechanical deformation

## Abstract

Silicon, being the fundamental material for modern semiconductor devices, has seen continuous advancements to enhance its electrical and mechanical properties. Strain engineering is a well-established technique for improving the performance of silicon-based devices. In this paper, we propose a simple method for inducing and permanently maintaining strain in silicon through pure physical bending. By subjecting the silicon substrate to a controlled bending process, we demonstrate the generation of strain levels that persist even after the removal of external stress, with a maximum strain value of 0.4%. We present a comprehensive study of the mechanics behind this phenomenon, a full finite element mechanical model, and experimental verification of the bending-induced strain in Si membranes using electron backscatter diffraction measurements. Our findings show the potential of this approach for strain engineering in high-performance silicon-based technologies without resorting to complex and expensive fabrication techniques.

## 1. Introduction

The importance of strain engineering in semiconductor materials has increased in recent years [1] as the demand for faster, more efficient, and high-quality hetero-structure electronic and photonic devices has grown [2]. Strain engineering can help improve the performance of devices in a number of ways [3,4,5], including increasing carrier mobility [6,7], enhancing transistor performance and improving device speed [8,9], reducing parasitic capacitance [10], changing the band gap [11,12], improving the refractive index [13] and improving signal propagation [14]. The benefits of strain engineering, combined with its compatibility with existing manufacturing processes, make it a promising technology to improve the performance of devices [15].

Traditional methods of inducing material strain involve the design of epitaxial growth processes in planar materials [16], ion implantation [17] and exploiting the mismatch between heteroepitaxial layers, e.g., the silicon and germanium lattices [18]. These methods, though well-developed, are generally limited to implementation on a single substrate. More recently, semiconductor nanomembranes (NMs) have emerged as a promising materials platform for strain engineering [19]. This platform offers unique opportunities for strain manipulation, both through spontaneous elastic strain upon NM release, and through the application of external mechanical stress [20].

Here, we propose a simple approach to induce and maintain strain in silicon through physically bending thin silicon membranes for strain determination. The controllable bending of the thin film membranes is achieved using simple topological features on the substrate and printing the membranes across these features using micro-transfer printing. A silicon membrane is fabricated by using standard nanofabrication techniques and transfer-printed on ridges by using elastomeric transfer printing [21]. The printed membrane conforms to the top of the ridge, resulting in physical bending. First, a simple modelling of the induced stress in membranes is established by treating them as cantilevers with fixed boundary conditions. This simple model is then compared to a full finite element mechanical model. To verify the simulation results, a transfer-printed membrane across a micron-scale ridge is analysed using electron backscatter diffraction (EBSD) measurements. We report that normal and shear strains of 0.4% and 0.2%, respectively, were achieved, showing the potential for future silicon-based device designs, where strain engineering can be efficiently integrated into the existing fabrication processes, paving the way for a new era of high-performance and energy-efficient electronic devices.

## 2. Transfer Printing of Silicon Membranes

### 2.1. Fabrication Processes

**Figure 1:**
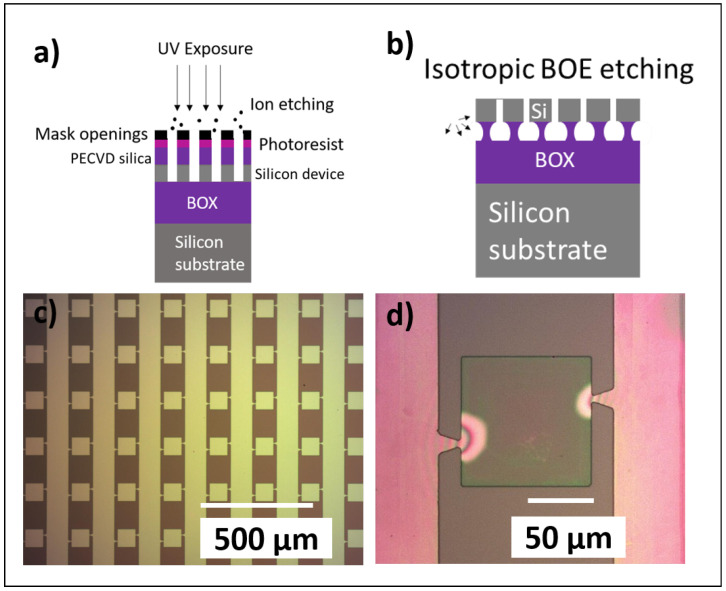
(**a**) Schematic representation of the basic fabrication steps of the silicon membranes, (**b**) schematic representation of the suspension of the silicon membranes, (**c**) fabricated array of silicon membranes before suspension, and (**d**) suspended silicon membrane, supported by the two antisymmetric triangular anchors on the sides.

A silicon-on-insulator (SOI) wafer (Ultrasil LLC, CA, US) was used with silicon thickness 1.5 μm and buried oxide (BOX) 5 μm. A blanket etch was performed on the top silicon layer using reactive ion etching in order to define the desired membrane thickness (in this case, 220 nm thickness). By using a standard lithography process, as shown in the schematic in Figure 1a, a square shape of the membranes was achieved with a size of 100×100 μm^2^, as shown in Figure 1c. In order to enable the release of the membranes from their native substrate, an under-etching process is required to etch the BOX underneath the Si membranes. In this work, a 7:1 ratio Buffered Oxide Etch (BOE, MicroChemicals GmbH) was used. BOE etches silicon dioxide isotropically with a rate of 80 nm/min, as is shown in the schematic in Figure 1b. After this final fabrication step, the membranes are in a suspended state to allow for their pick-up and subsequent placement. A micrograph of a representative suspended membrane is shown in Figure 1d.

The transfer printing process uses an elastomeric polymer, polydimethylsiloxane (PDMS, Dow Corning, MI, US), as a manipulation tool (thereafter referred to as stamp) to pick up single membranes from their native substrate (donor) and place them on the target substrate (receiver). The adhesive properties of the PDMS were controlled using the mixing ratio between the monomer base and the curing agent. In this case, a 10:1 ratio stamp was used. For better adhesion, the stamp had the same lateral dimensions as the membranes, ensuring that the applied force was evenly distributed, maximizing the effective contact area and minimizing the possibility of air gaps or partial contact; its thickness was typically 100 μm. For better release, five pyramidal features with a height of 7 μm were present on the corners and in the middle of the stamp. The stamp was fabricated using liquid casting onto a mold, which was fabricated on a silicon wafer. Standard lithography and anisotropic chemical etching processes were used to define the pyramidal features. The shaping was achieved using a hot potassium hydroxide (KOH) wet etching process, performed at 85 °C with a dilution ratio of 0.665 g/mL in deionized (DI) water. A thick negative tone SU8-5 photoresist was subsequently patterned using photolithography to determine the height of the stamp. The PDMS was then poured into the mold and cured for 24 h at room temperature, followed by 1 h on a hot plate at 80 °C. After the curing process, the PDMS stamp was released from the mold and mounted in the transfer printing system [22].

The ridges on the receiver substrate were made of an SU8-5 negative tone photoresist (Kayaku Advanced Materials, MA, US) and fabricated on silicon substrate by using laser lithography. By adjusting the photoresist thickness and the laser direct writing parameters, the height and width of the fabricated features were varied. In particular, in this study, the ridge had a height of *h* = 1.50 μm and a width of *w* = 3.5 μm.

### 2.2. Printed Membrane Optical Analysis

**Figure 2:**
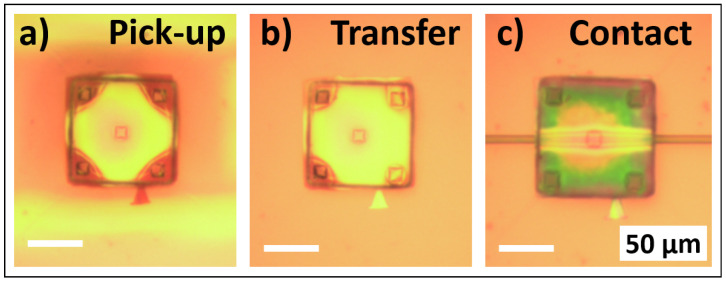
Illustration of the stamp transfer printing. (**a**) The membrane is picked from the host substrate; (**b**) the membrane is attached to the stamp and transferred to the receiver substrate; and (**c**) the membrane is brought into contact with the ridge.

**Figure 3:**
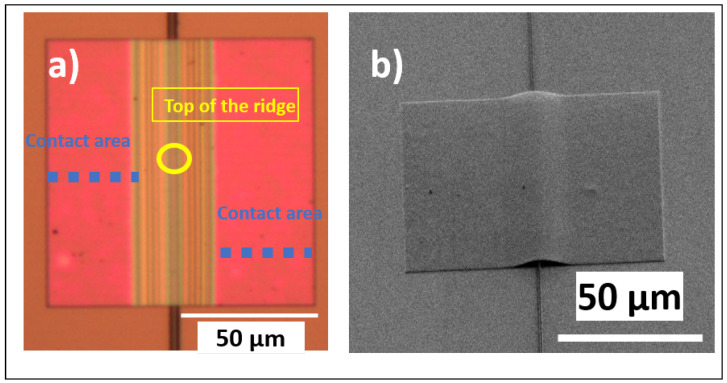
Transfer-printed membrane on ridge (**a**) top-view optical micrograph at a 50× magnification (the blue dotted line indicates the contact area and the yellow circle indicates the top of the ridge) and (**b**) SEM image at a tilt angle of 35° (tilt-corrected).

The fabricated silicon membranes were picked up from the donor and printed on the silicon substrate on the ridge using transfer printing with a controlled reversible adhesion mechanism. The process of conformal printing to existing features on the receiving substrate is well documented in previous publications [23,24]. The whole process relies on the competitive adhesion between different materials, bringing them into contact through Van der Waals forces. This process is rate-sensitive, which means that we can enhance the pick-up and printing by adjusting the speed of the process. The membrane was picked up with high speed from the donor and transfer-printed with low speed in the middle of the ridge, with micrometric accuracy. An illustration of the process can be seen in Figure 2. The stamp is brought into close contact with the membrane and by quickly pulling it, due to the preferable adhesion, the membrane is attached to the stamp (Figure 2a). The stamp is moved on top of the receiver substrate (Figure 2b) and brought into contact with the ridge (Figure 2c). By slowly removing the stamp, the membrane is finally printed on top of the ridge. An optical micrograph and a scanning electron microscopy (SEM) image at a tilt angle of 35° of the printed membrane on top of the ridge are shown in Figure 3a and Figure 3b, respectively.

The printed membrane shows its clear conformation to the ridge it is printed over; hence, a mechanical deformation occurs that is permanent once the stamp has released the membrane in place. In the optical micrograph of Figure 3a, optical fringes are easily identifiable. They originate from the optical interference of the illumination light due to the gap between the back of the membrane and the substrate. In addition, the width of these fringes increases with the increase in distance from the top of the ridge, indicating a non-uniform gap, i.e., a curvature of the membrane. The areas on both sides of the membrane where there are no fringes correspond to full contact with the receiving substrate. Knowing the distance-per-pixel calibration of the imaging setup, the contact length can thus be estimated as 35 μm. The repeatability of the measurement to estimate the contact length is related to the pixel resolution of the microscope photos, which was calculated as 6.8 pixels/μm, providing a resolution of 150 nm/pixels. The tilted SEM imaging in Figure 3b visually confirms the previous description, namely, an s-shape conformation behaviour.

## 3. Theoretical Analysis of Membranes’ Mechanical Deformation

### 3.1. Analytical Analysis

**Figure 4:**
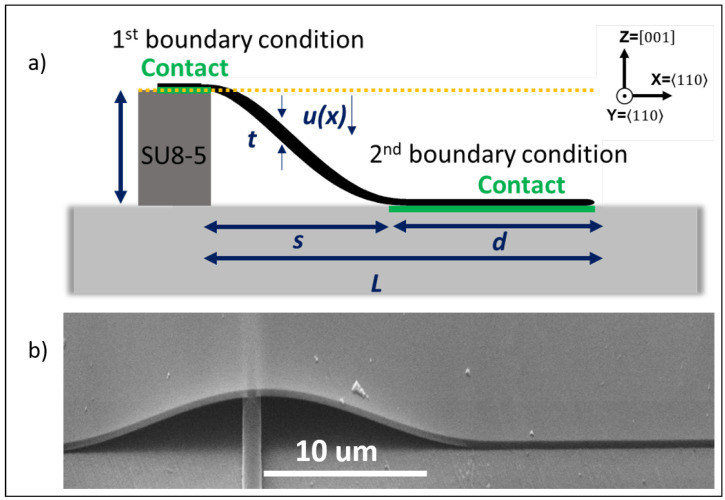
(**a**) Schematic representation of the s-shape cantilever beam, showing the contact length *d*, the unadhered length *s*, the thickness *t*, the deformation equation u(x), and the height of the ridge (*h*). (**b**) SEM image at a tilt of 70° of the transfer-printed membrane on top of the ridge.

The printed membrane on top of the ridge creates an s-shape conformal behavior on each side, which was previously studied to measure the adhesion of cantilevers [25,26]. In this section, the deformation in the membrane of the cantilever is thus analytically studied to verify if the s-shape analytical solution is suitable. The general formalism is provided by the following equation:

(1) $$u\left(x\right)=h{\left(x/s\right)}^{2}\left(3-2\left(x/s\right)\right)$$

where u(x) is the equation that provides the deformation as a function of *x*, *h* is the height of the support, and *s* is the unadhered part [25,26]. The unadhered length can be easily calculated by subtracting the contact area from the total length, (L−d). Figure 4a shows a schematic representation of the s-shape deformation related to Equation (1). Figure 4b shows an SEM image at a tilt of 70° of the membrane, showing a clear curvature with radius *R* on top of the ridge. In association with a simplified version of the bending theory, the normal uniaxial strain in the perpendicular direction of deformation can also be derived. The first derivative, $u^{\prime }\left(x\right)$, provides the slope of the beam and is related with the angle of curvature θ through the following equation:

(2) $$\tan\theta =du\left(x\right)/dx=u^{\prime }\left(x\right)$$

The second derivative, $u^{\prime \prime }\left(x\right)$, represents the curvature of the beam. It provides information about how the slope is changing along the beam and is given by the following equation:

(3) $$\kappa =\frac{d^{2}u\left(x\right)}{dx^{2}}=\frac{6h}{s^{2}}-\frac{12h}{s^{3}}x$$

The radius of curvature *R* is defined as the reciprocal of the curvature κ:

(4) R=1/κ

By using Equations (3) and (4), we can derive the following equation for the radius of curvature:

(5) $$R=\frac{{\left(1+u^{\prime 2}\right)}^{3}/2}{u^{\prime \prime }}$$

which relates the first and second derivatives of the deformation with the radius. By differentiating Equation (1), the radius of curvature of the deformed membrane is calculated along with the strain value using the simple equation of bending theory:

(6) ε=δR/R=t/2R

where ε is then defined as the ratio of the change in radius length to the original radius length and *t* is the thickness of the membrane.

The analytical model employed in this study is based on linear bending theory and assumes small deformations, which simplifies the problem and provides clear physical insights. However, at strain levels approaching 0.5%, these assumptions may become less accurate due to limitations regarding the assumptions of material linearity in the model. To address this limitation and ensure a more comprehensive understanding of the mechanical behaviour, a finite element analysis (FEA) model was also developed. The FEA approach allows for the inclusion of nonlinear effects and more complex boundary conditions, offering improved accuracy in predicting strain distributions, particularly in regions of high deformation or near critical features such as contact interfaces. This combined modelling strategy ensures both analytical clarity and numerical robustness across the full range of deformation being studied.

### 3.2. Finite Element Analysis

The mechanical deformation simulations performed here employ the ANSYS Mechanical (Version 2022 R2) software [27,28] to perform an FEA analysis of thin-film silicon cantilevers. These cantilevers were arranged so that one end was supported by a ridge with a height denoted as ‘*h*’, while the other end bent downward to attach to the substrate. As a result, the membrane formed an s-shaped deformation characterized by two opposing curvatures along the membrane. Investigating the deflection and curvature of this deformation yields valuable insights into the induced strain.

**Figure 5:**
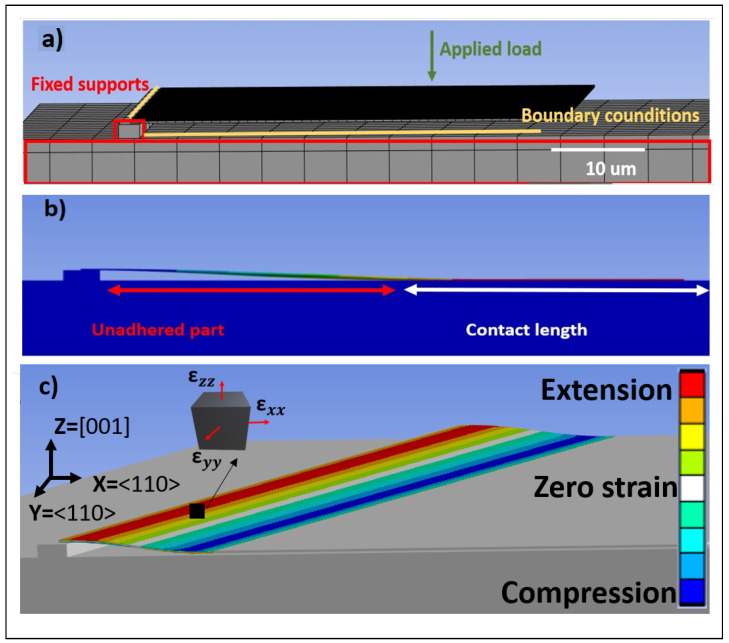
Ansys model for the deformed membrane. (**a**) Initial state of the model, highlighting the fixed supports (ridge and substrate), the applied downwards force (Z direction), (**b**) the shape after the simulation (s-shape deformation), and (**c**) the strain components of the deformed membrane with respect to the reference system.

In the FEA process, material properties must be defined before any geometry design is implemented. In this study, silicon was used as the material, with the following properties: a density (*p*) of 2330 kg/m^3^, isotropic elasticity with a Young’s modulus of 165 GPa, a Poisson’s ratio of 0.22, a bulk modulus of 9.821 × $10^{10}$ Pa, and a shear modulus of 6.76 × $10^{10}$ Pa. The model has two fixed, rigid boundary conditions, one between the top of the ridge and the bottom surface of the membrane, and the second on the surface of the substrate, representing the contacted area. In this model, adhesion is represented as a vertically applied distributed force to the bottom surface of the membrane, which could be varied as a parameter, as shown in Figure 5a) and simplifies the complex interaction between the membrane and substrate. This idealization was chosen for its ability to approximate the net adhesive forces, such as van der Waals attraction, while maintaining its computational efficiency. Although this representation does not capture the full complexity of the adhesion mechanism, it sufficiently models the overall effect on the membrane’s deformation. This approach was found to be effective in reproducing the experimental strain distributions, where the adhesion is observed to be relatively uniform and does not cause significant lateral movement or delamination of the membrane. More detailed adhesion models could be employed for higher fidelity, but the current simplification provides a reasonable balance between model complexity and accuracy in the system under study. The constraint at the top of the ridge causes the membrane’s farthest point from the ridge to make initial contact with the substrate. As the applied force increases, the contact area between the membrane and the substrate expands. In this way, the adhesion force between the bottom surface of the membrane and the substrate is modelled and can then be compared with the experimental results through the measured contact length *d* of the two surfaces, as described in the schematic of Figure 5b.

The behavior of the cantilever under variable loads acting downwards in the Z-direction is systematically modelled with a magnitude in the order of μN. The dimensions of the cantilever are 50×100 μm^2^ with a thickness of 220 nm. Figure 5b shows the resulting shape of the cantilever after the simulation and shows the top surface sampling of strain across one side of the membrane from ridge to the contact area. This will give the following strain components, as can be seen on the element in Figure 5b. The ridge height was set at 1.50 μm, matching the experimental value. Under these different values, the curvature and the s-shape of the cantilever beam changed significantly, contributing to the different normal ($\varepsilon_{\mathrm{xx}}$ and $\varepsilon_{\mathrm{zz}}$) and shear ($\varepsilon_{\mathrm{xy}}$) strains in the model, as analysed further below.

**Figure 6:**
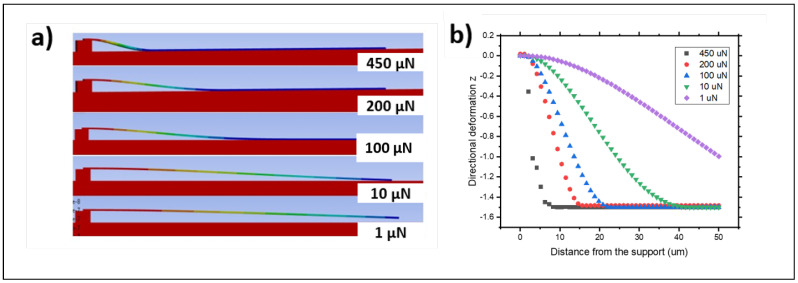
(**a**) Representative s-shaped deformations for silicon membranes with various applied vertical load magnitude in relation to the contact length. (**b**) Vertical deformation (Z direction) with the distance from the support for selected vertical load magnitudes in the μN range.

Figure 6a shows the simulated results of the membrane deformation under selected magnitudes of the applied force of 1, 10, 100, 200 and 450 μN. The membrane starts contacting the substrate for loads in the region of 10 μN, while a load of magnitude of 1 μN is not sufficient to bend the membrane into contact with the surface underneath. Figure 6b shows the actual membrane profiles for the selected load magnitudes and depicts the associated vertical displacement against the contacted area. Upon increasing the magnitude of the applied load, the overall shape of the deformed membrane changed in terms of curvature, as well as contacted and unadhered lengths.

### 3.3. Comparison of Numerical Model Results

**Figure 7:**
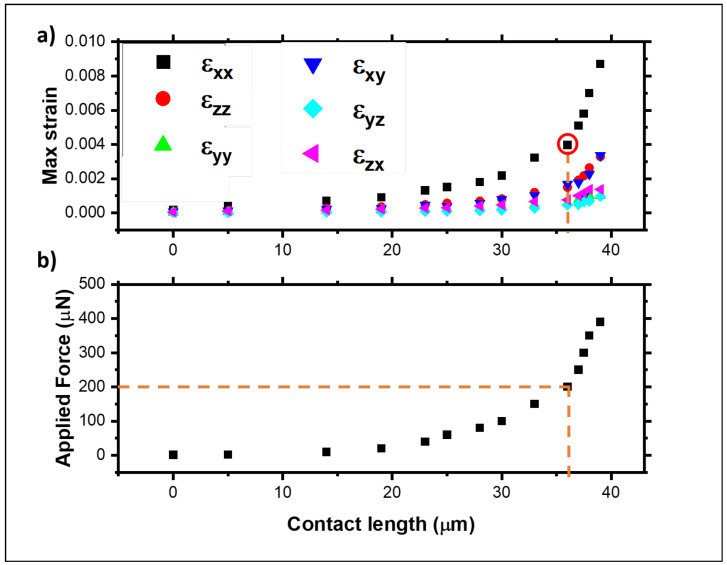
(**a**) Maximum normal and shear strains against the contact length and (**b**) the applied vertical force against the contact length for the ridge and membrane thickness corresponding to the experimental case: *h* = 1.50 μm and *t* = 220 nm, respectively. The red circle indicates the matching contact length with the measured value.

**Figure 8:**
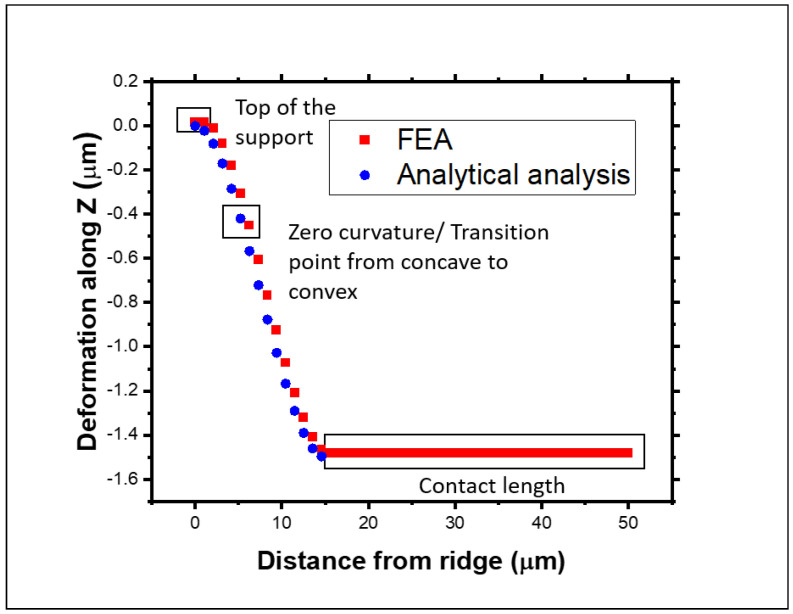
Comparison of the mechanical deformation in the membrane obtained from the analytical and FEA models with the experimental dimensions as parameters.

The top panel of Figure 7 shows the maximum values of the shear and normal strains for each contact length in the FEA model, showing that the strain is strongly related to the contact length and, consequently, to the final shape induced by the different vertical loads, as expected. Similarly, the bottom panel of Figure 7 shows the variation in the part (contact length) of the membrane that adhered to the substrate while the vertical load was varied. The contact length shows a sharp increase with loads between 0 and 150 μN, with the contact length increasing from 0 to about 30 μm. At higher loads, the contact length plateaus at around 40 μm. For loads above 400 μN, there is a sudden increase in the contact length to above 45 μm, which indicates where the model fails. Considering the experimental results from the previous section of a contact length of 35 μm (marked with a dashed line in both panels of Figure 7), the FEA model indicates that the membrane has an equivalent applied load of 200 μN. This corresponds to the maximum normal strain across the whole membrane of 4×$10^{-3}$, 0.6×$10^{-3}$, and 1.5×$10^{-3}$ for $\varepsilon_{\mathrm{xx}}$, $\varepsilon_{\mathrm{yy}}$, and $\varepsilon_{\mathrm{zz}}$, respectively, and a shear of 1.8×$10^{-3}$, 0.5×$10^{-3}$, and 0.8×$10^{-3}$ for $\varepsilon_{\mathrm{xy}}$, $\varepsilon_{\mathrm{yz}}$ and $\varepsilon_{\mathrm{zx}}$, respectively, with an error arising from the mesh size, in the order of $10^{-6}$. Figure 8 shows a comparison between the results obtained from the analytical model and the FEA models, plotted for a vertical load of 200 μN, using the experimental case’s parameters. Both models provide sensible and similar results in terms of the s-shape deformation of the membrane. The directional deformation in the Z-direction allows for the identification of particular points of interest. The top, flat part of the graph with zero deformation refers to the point at the top of the ridge, while between 0 and 15 μm, the membrane does not adhere to the substrate. In the middle of the unadhered part, there is a transition point from concave to convex, where the curvature is zero. As the deformation reaches a height of −1.50 μm, the membrane makes contact with the substrate that corresponds to the start of the contact length.

**Figure 9:**
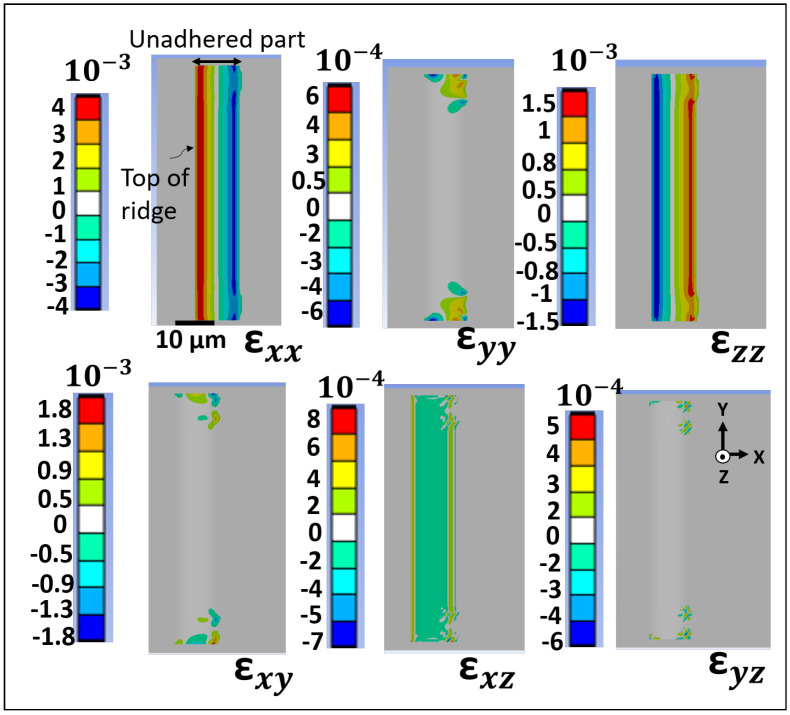
Normal ($\varepsilon_{\mathrm{xx}}$, $\varepsilon_{\mathrm{yy}}$, $\varepsilon_{\mathrm{zz}}$) and shear ($\varepsilon_{\mathrm{xy}}$, $\varepsilon_{\mathrm{xz}}$, $\varepsilon_{\mathrm{yz}}$) strain maps obtained from finite element simulations.

**Figure 10:**
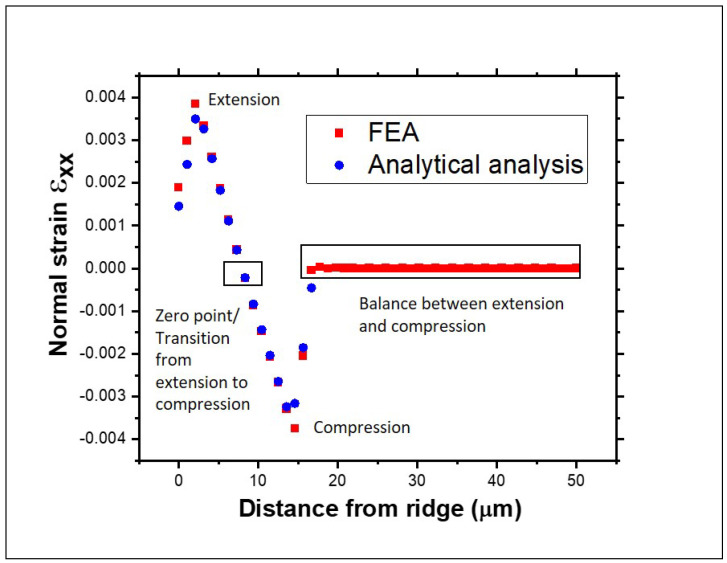
Comparison between the normal $\varepsilon_{\mathrm{xx}}$ strain obtained from the simulations and the theoretical model.

The normal and shear strain maps obtained from the FEA simulations are shown in Figure 9. The simulations show that the normal strains $\varepsilon_{\mathrm{xx}}$ and $\varepsilon_{\mathrm{zz}}$ are the largest. The shear strain is mainly induced in the XY plane, while the shear strains in the XZ and YZ planes are very small. The strain gradient across the YY, XY, and YZ directions is most pronounced at the edges of the membrane. This effect is due to the boundary constraints of the simulation model, where the midpoint cross-section perpendicular to the ridge is fixed, restricting displacement in these directions, particularly in the central region. The following section will further analyze the shear strain components, focusing on the middle part of the membrane. Taking the line scans from the FEA simulation and the analytical model, each strain component can be plotted as a function of the distance from the support. Figure 10 shows the strain value for the $\varepsilon_{\mathrm{xx}}$ component, which is the dominant one for both models. The strain values for the analytical model were obtained from Equations (5) and (6), with an error in the order of $10^{-5}$.

It is noticeable that the results from the FEA simulations match very well the predicted values from the analytical formalism. In Figure 10, the areas of interest are highlighted. The strain values go from extension to compression, passing through a zero point which indicates this transition. This is also the point where the curvature equals zero, as previously described. The zone with a constant strain of zero corresponds to the contact length where there is complete balance between extension and compression.

## 4. Electron Backscatter Diffraction Measurements

### 4.1. Radius of Curvature Assessment

In order to study the resultant induced strain on the printed cantilevers, we performed electron backscatter diffraction (EBSD) measurements. The EBSD measurements were carried out in a variable-pressure field-emission gun electron microscope (FEI Quanta 250, FEI Technologies Inc. OR, USA) using an Oxford Instruments Nordlys EBSD system. After initial indexing (Oxford Instruments Refined Accuracy method), refined orientation information and strain determination were carried out via cross-correlation of the experimental electron backscatter pattern with dynamically simulated patterns [29,30,31]. Strain distorts the crystal lattice, and since an EBSD pattern is a two-dimensional projection of the lattice, strain also distorts the pattern. Comparing the experimental patterns with dynamically simulated patterns from the unstrained Si lattice allows for the determination of the six components of the relative deformation gradient tensor with a misorientation resolution in the order of 0.006° and a relative strain resolution in the order of 10^−4^ [32]. Afterwards, the EBSD data were analysed using MTEX, a Matlab-based toolbox [33]. The EBSD data were acquired at a sample tilt of 70° and electron beam voltage of 30 kV; the pattern size was 1344×1024 px^2^ and the step size was 500 nm.

**Figure 11:**
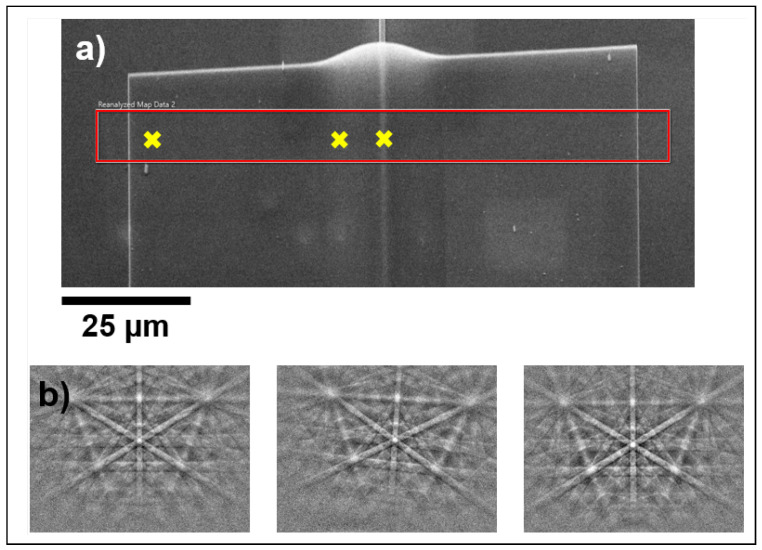
(**a**) SEM image of the Si membrane measured using EBSD. The measured area is marked by the box. (**b**) Example electron backscatter patterns acquired from the flat left side of the Si membrane, the side, and the top. The spots are marked by the crosses in the SEM image in (**a**).

**Figure 12:**
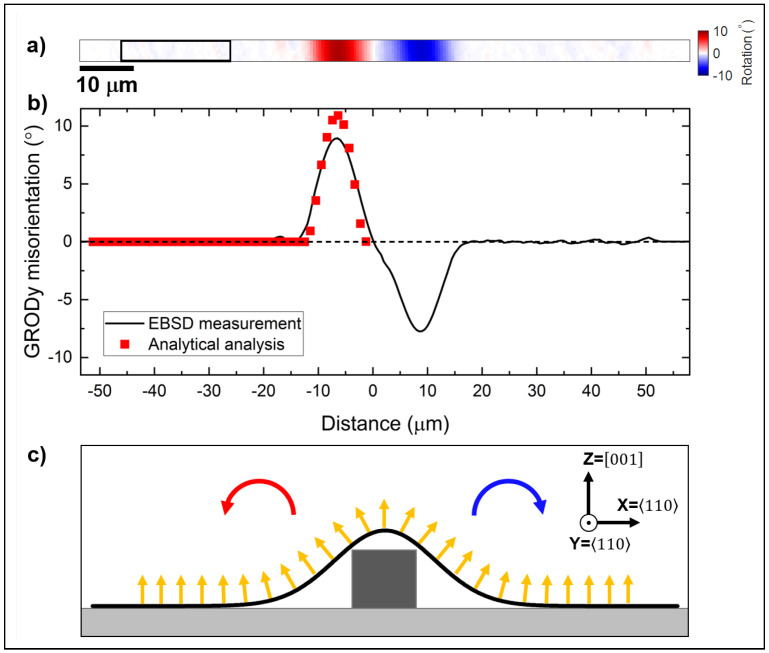
(**a**) Misorientation angle around the Y axis (GRODy). The black box on the left marks the misorientation reference. (**b**) GRODy line scan across the Si membrane. The line scan was averaged over the entire height of the map. The angle of curvature from the analytical analysis is superimposed. (**c**) Cross-section schematic of the membrane showing the rotation around the Y axis.

Figure 11a shows an SEM image of the Si membrane that was investigated using EBSD. The investigated area is delimited by the red rectangle and three example EBSD patterns marked by crosses are shown in Figure 11b. The electron backscatter pattern on the sloped side of the membrane (middle) shows considerable rotation compared with the patterns from the flat side (left) and top of the ridge (right). This relates to a change in the crystallographic orientation of the Si lattice. An EBSD pattern is a two-dimensional projection of the crystal lattice. Therefore, when the lattice rotates, the pattern rotates. This can be observed on the slope of the membrane where the membrane is bent around the waveguide, inducing a rotation of the lattice compared with the top and flat side. The variation in the orientation of the crystal lattice of the membrane can be assessed through comparing how much the orientation in each pixel in the dataset deviates from a chosen reference orientation. This is referred to as grain reference orientation deviation (GROD) analysis. The Si membrane is folded over the ridge, which is parallel to the Y axis in our reference in the analysis, the orientation change should only be caused by a rotation around the Y, while rotations around the X and Z axis (GRODx and GRODz, respectively) are negligible. Figure 12a shows the misorientation around the Y axis (GRODy) for the Si membrane, where the reference orientation is the mean orientation on the flat adhered region of the membrane, as marked by the box in the figure.

An averaged line scan extracted from the GRODy map is shown in Figure 12b. A symmetrical orientation change is clearly noticeable on either side of the top of the membrane. Due to the symmetrical orientation change around the top of the membrane, the rotation is positive to one side and negative to the other side, as illustrated in Figure 12c. The maximum misorientation was measured to be about 8–9° ± 0.006°. The angle of the membrane curvature, as determined from the analytical model, is also plotted in Figure 12b. It agrees reasonably well with the EBSD measurement, although it estimates a maximum misorientation in the membrane of 10.5° ± 0.007°. This discrepancy is attributed to the simplifications of the analytical model.

### 4.2. Normal Strain Assessment

**Figure 13:**
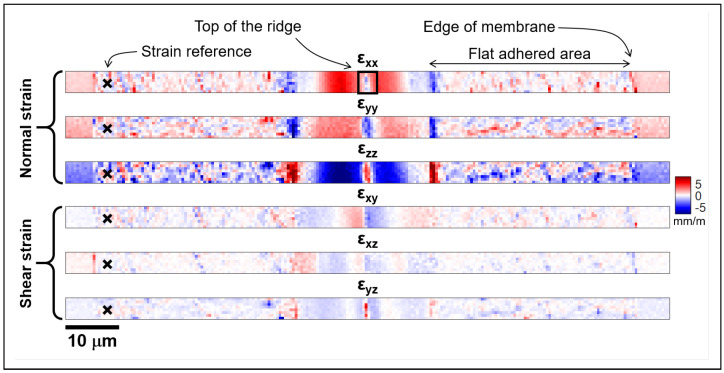
Normal ($\varepsilon_{\mathrm{xx}}$, $\varepsilon_{\mathrm{yy}}$, $\varepsilon_{\mathrm{zz}}$) and shear ($\varepsilon_{\mathrm{xy}}$, $\varepsilon_{\mathrm{xz}}$, $\varepsilon_{\mathrm{yz}}$) strain maps obtained from the EBSD measurements. The black cross on the left marks the strain reference.

The strain maps were also obtained from the EBSD measurements. Figure 13 shows the normal and shear strains inferred from the rectangular area marked on the SEM image in Figure 11a. As seen previously in the FEA model, the dominant strain is present in the $\varepsilon_{\mathrm{xx}}$ and $\varepsilon_{\mathrm{zz}}$ components, with values as high as 5 × $10^{-3}\pm 10^{-4}$.

**Figure 14:**
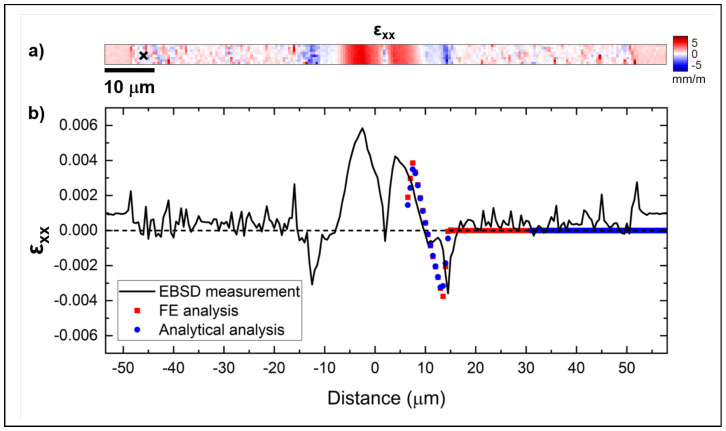
(**a**) $\varepsilon_{\mathrm{xx}}$ normal strain map obtained from the EBSD measurement and (**b**) linescan of the $\varepsilon_{\mathrm{xx}}$ component across the Si membrane. The line scan was averaged over the entire height of the map. The $\varepsilon_{\mathrm{xx}}$ values from the FE and analytical analysis are superimposed.

An averaged line scan extracted from the normal strain map of the $\varepsilon_{\mathrm{xx}}$ component in Figure 14a is shown in Figure 14b, along with the equivalent data estimated with the analytical and FEA models. The predicted values in both models correspond reasonably well with the EBSD measurement. From the top down, an extension (tensile strain) is shown, passing through a transition point to a region of contraction, where the membrane is compressed before approaching zero at the contact area. The measured values are slightly less symmetric around the transition point. Effectively, the Si membrane around the top ridge area is not perfectly flat, and this curvature deviates from the assumption that both models make perfect contact with the ridge. Finally, in the experimental case, the sides of the membrane may not be perfectly parallel to the ridge, which will skew the strain line scan. The offset in the measured strain values, in the order of ±0.001, can be attributed to several differences between the idealized assumptions in the models, the physical fabrication process, and the experimental conditions. Firstly, the analytical model and FEA simulations assume perfect geometry, material homogeneity, and ideal boundary conditions, which may not fully represent the real-world complexities of the membrane. Small imperfections in the model geometry, such as slight deviations from flatness, or variations in how the membrane is supported, can contribute to the offset. Additionally, the assumption of zero initial strain in the silicon membrane may not be accurate, as residual stresses from the fabrication process, such as deposition, etching, or thermal treatments, can introduce non-zero initial strain. Furthermore, thermal effects during the experiment or in the fabrication process can cause strain due to temperature variations, especially given the membrane’s thickness. Moreover, surface roughness and microstructural variations on the membrane can impact the strain measurements, particularly in EBSD, where the electron beam interacts differently with surfaces of varying roughness. The transfer printing of silicon membranes on top of a ridge or equivalent waveguide demonstrates that a strain of about 0.004 ± 0.0001 can be induced. By comparing this value with the literature, we find that it is within a region that can significantly change the silicon band gap by a value as high as 0.10 eV [34].

## 5. Conclusions

In this work, it was shown that a strain modification can be induced on a silicon membrane by physically bending it through a transfer-printing process over the features present on the target substrate. The incurred deformation remains once the external force applied during the transfer is removed. We performed analytical and FEA simulations of the mechanical deformation and compared the results obtained in both models to the experimental values taken from an EBSD measurement. These results show that the simple analytical bending model is a good approximation for modelling this type of membranes. The changes in crystal orientation reflecting the conforming behavior of the printed membrane and normal strain was assessed at 0.4% in the measurement and with the models. This strain corresponds to a band gap change of 0.10 eV. This printing method can be employed to locally modify the properties and functionality of silicon-based photonic devices in a heterogeneous integration paradigm.

## Data Availability

The data associated with this research are available at https://doi.org/10.15129/4435963e-15e0-4bd0-84ff-3c69e002ea01 or from the corresponding author.

## Abbreviations

The following abbreviations are used in this manuscript:

Si	Silicon
NMs	Nanomembranes
EBSD	Electron Backscatter Diffraction
SOI	Silicon-on-insulator
BOX	Burried Oxide
BOE	Buffered Oxide Etch
PDMS	Polydimethylsiloxane
KOH	Potassium hydroxide
DI	Deionized water
SEM	Scanning Electron Microscopy
FEA	Finite Element Analysis
GROD	Grain Reference Orientation Deviation
